# Contributions of the Sodium Leak Channel NALCN to Pacemaking of Medial Ventral Tegmental Area and Substantia Nigra Dopaminergic Neurons

**DOI:** 10.1523/JNEUROSCI.0930-22.2023

**Published:** 2023-10-11

**Authors:** Dana E. Cobb-Lewis, Lorenzo Sansalone, Zayd M. Khaliq

**Affiliations:** ^1^Cellular Neurophysiology Section, National Institute of Neurological Disorders and Stroke, National Institutes of Health, Bethesda, Maryland 20892; ^2^Institute for Neuroscience, George Washington University School of Medicine and Health Sciences, Washington, DC 20037

**Keywords:** action potential, dopamine, pacemaking, sodium leak current, NALCN

## Abstract

We tested the role of the sodium leak channel, NALCN, in pacemaking of dopaminergic neuron (DAN) subpopulations from adult male and female mice. In situ hybridization revealed NALCN RNA in all DANs, with lower abundance in medial ventral tegmental area (VTA) relative to substantia nigra pars compacta (SNc). Despite lower relative abundance of NALCN, we found that acute pharmacological blockade of NALCN in medial VTA DANs slowed pacemaking by 49.08%. We also examined the electrophysiological properties of projection-defined VTA DAN subpopulations identified by retrograde labeling. Inhibition of NALCN reduced pacemaking in DANs projecting to medial nucleus accumbens (NAc) and others projecting to lateral NAc by 70.74% and 31.98%, respectively, suggesting that NALCN is a primary driver of pacemaking in VTA DANs. In SNc DANs, potentiating NALCN by lowering extracellular calcium concentration speeded pacemaking in wildtype but not NALCN conditional knockout mice, demonstrating functional presence of NALCN. In contrast to VTA DANs, however, pacemaking in SNc DANs was unaffected by inhibition of NALCN. Instead, we found that inhibition of NALCN increased the gain of frequency-current plots at firing frequencies slower than spontaneous firing. Similarly, inhibition of the hyperpolarization-activated cyclic nucleotide-gated (HCN) conductance increased gain but had little effect on pacemaking. Interestingly, simultaneous inhibition of NALCN and HCN resulted in significant reduction in pacemaker rate. Thus, we found NALCN makes substantial contributions to driving pacemaking in VTA DAN subpopulations. In SNc DANs, NALCN is not critical for pacemaking but inhibition of NALCN makes cells more sensitive to hyperpolarizing stimuli.

**SIGNIFICANCE STATEMENT** Pacemaking in midbrain dopaminergic neurons (DAN) relies on multiple subthreshold conductances, including a sodium leak. Whether the sodium leak channel, NALCN, contributes to pacemaking in DANs located in the VTA and the SNc has not yet been determined. Using electrophysiology and pharmacology, we show that NALCN plays a prominent role in driving pacemaking in projection-defined VTA DAN subpopulations. By contrast, pacemaking in SNc neurons does not rely on NALCN. Instead, the presence of NALCN regulates the excitability of SNc DANs by reducing the gain of the neuron’s response to inhibitory stimuli. Together, these findings will inform future efforts to obtain DAN subpopulation-specific treatments for use in neuropsychiatric disorders.

## Introduction

Dopaminergic neurons (DANs) of the substantia nigra pars compacta (SNc) and ventral tegmental area (VTA) fire action potentials spontaneously in a rhythmic fashion (“pacemaking”), resulting in sustained dopamine release necessary for behavior (Grace and Bunney, 1984; Johnson and North, 1992; Nedergaard and Greenfield, 1992). Subpopulations of DANs send projections to a variety of brain regions. SNc DANs project mainly to the dorsal striatum, while VTA DANs project to either the lateral shell (latNAc), medial shell (medNAc), or core (NAcc) of the nucleus accumbens (NAc) (Ikemoto, 2007; Aransay et al., 2015; Beier et al., 2015; Lerner et al., 2015; Poulin et al., 2018). In addition, DAN subpopulations differ in their intrinsic properties and ionic conductances that support firing (Margolis et al., 2006; Lammel et al., 2014; Baik, 2020). To understand how DAN subpopulations contribute to behavior and how to target specific DAN cell populations for treatments in disease states will require better knowledge of the ionic mechanisms that underlie spontaneous action potential firing in DAN subpopulations.

Multiple subthreshold conductances contribute to pacemaking and this mixture of ion channels differs among midbrain DAN subpopulations. For example, “conventional” DANs located in the SNc exhibit large hyperpolarization-activated cyclic nucleotide-gated (HCN) conductances (Mercuri et al., 1995; Neuhoff et al., 2002), and pacemaking in these cells involves a prominent interspike current from low-threshold calcium channels (Kang and Kitai, 1993; Puopolo et al., 2007; Guzman et al., 2009; Hage et al., 2016) as well as persistent voltage-gated sodium conductances (Puopolo et al., 2007; Philippart et al., 2016). By contrast, VTA DANs exhibit less reliance on voltage-gated calcium conductances, calcium-dependent conductances (Koyama et al., 2005; Khaliq and Bean, 2010; Philippart et al., 2016) and HCN conductances (Neuhoff et al., 2002; Lammel et al., 2008; Kramer et al., 2018). Instead, a sodium leak conductance is the dominant inward current at subthreshold voltages in the VTA (Khaliq and Bean, 2010). However, the molecular correlate of this current is unknown, and whether its contribution to firing differs among DAN subpopulations is incompletely understood.

NALCN is a tetrodotoxin (TTX)-insensitive sodium leak channel expressed ubiquitously throughout the brain and spinal cord shown to drive pacemaking in neurons of the substantia nigra pars reticulata, suprachiasmatic nucleus, and brain respiratory regions, suggesting a general role for NALCN in driving pacemaking activity (Flourakis et al., 2015; Lutas et al., 2016; Shi et al., 2016; Yeh et al., 2017; Ford et al., 2018). However, studies examining the role of NALCN in pacemaking of SNc DANs have been conflicting. Past work in a mouse line in which NALCN is conditionally knocked out from birth (NALCN cKO) suggested that NALCN plays a strong role in pacemaking (Philippart and Khaliq, 2018). Experiments testing pharmacological blockade of NALCN have observed opposite effects in SNc, showing either complete inhibition of pacemaking (Um et al., 2021) or no effect on pacemaking (Jehasse et al., 2021). In VTA DANs, however, the role of NALCN in pacemaking has not yet been explored.

Here, we used calcium imaging, electrophysiology, and NALCN cKO mice to determine the contribution of NALCN to pacemaking of midbrain DAN subpopulations defined by their anatomical location or axonal projection targets. We show that the contribution of NALCN to pacemaking varies across DAN subpopulations along the medial-lateral axis. Pacemaking of the most medial VTA DANs projecting to the medNAc relies most strongly on NALCN, while pacemaking of lateral VTA DANs projecting to the latNAc relies less on NALCN. In SNc DANs, inhibition of NALCN has no effect on the rate of spontaneous firing. However, at rates that are slower than that of natural pacemaking, we found inhibition of NALCN increases the gain of the frequency-current curve, suggesting NALCN supports the slowest tonic firing rates in SNc DANs but is not critical for pacemaking. Thus, we demonstrate that the contributions of NALCN to pacemaking differ among DANs and these data lay the groundwork for DAN subpopulation-specific treatment of neuropsychiatric conditions.

## Materials and Methods

### Animals/subjects

All procedures were conducted in accordance with the guidelines established by the Animal Care and Use Committee for the National Institute of Neurologic Disorders and Stroke and the National Institutes of Health.

Adult (average age: 8.73 ± 0.87 weeks) male and female mice were used for electrophysiology, two-photon calcium imaging, and fluorescent ISH experiments. *DAT-Cre/Ai9 (SJL-Slc6a3(tm1.1(cre)Bkmn/J*, The Jackson Laboratory, catalog #006660) and *DAT-Cre/GCaMP6 (Ai95-RCL-GCaMP6f-D (Cg-Gt(ROSA)26Sor(tm95.1(CAG-GCaMP6f)Hze)/MwarJ*, The Jackson Laboratory, catalog #028865) mice were used as controls (WT) in experiments and compared with *NALCN conditional KO (NALCN cKO; NALCN^flox/flox^/DAT-Cre;C57BL/6 background*) mice to account for known differences in dopamine signaling and behavior in Dat-Cre expressing mice (Costa et al., 2021). NALCN cKO mice that lack expression of NALCN in DANs were generated as previously described (Philippart and Khaliq, 2018).

### Stereotaxic injections

All stereotaxic injections were performed using a Stoelting QSI (catalog #53311). Mice were anesthetized using 3% isoflurane and level of anesthesia was monitored throughout the surgery with 1.5% isoflurane.

The AAV1.Syn.Flex.GCaMP6f.WPRE.SV40 (Penn Vector Core) was injected bilaterally into the SNc (AP: −2.3; ML: ±1.6; DV: −5.3) or VTA (AP: −2.9; ML: ±0.4; DV: −4.9) of DAT-Cre and/or NALCN cKO mice with a Hamilton syringe (0.5 µl per side; 0.3 µl/min). Mice were used *ex vivo* for electrophysiology or calcium imaging 3-5 weeks after injection.

The retrograde labeler cholera-toxin subunit B (CTb) conjugated to Alexa-555 (CTb-AF555) was bilaterally injected into the medNAc (AP: 1.7; ML: ±0.5; DV: −4.6) or latNAc (AP: 1.2; ML: ±1.9; DV: −4.9) of Dat-Cre mice with a Hamilton syringe (0.2 µl per side; 0.3 µl/min). Mice were used *ex vivo* for electrophysiology 3-5 d after injection.

### Slice preparation

Mice were anesthetized with isoflurane, decapitated, and brains extracted. Coronal midbrain slices (200 µm) were prepared using a vibratome (Leica VT1200S). Slices were cut in ice-cold, oxygenated, glycerol-based slicing solution containing the following (in millimolar (mM)): 198 glycerol, 2.5 KCl, 1.2 NaH_2_PO_4_, 20 HEPES, 25 NaHCO_3_,10 glucose, 10 MgCl_2_, 0.5 CaCl_2_, 5 Na-ascorbate, 3 Na-pyruvate, and 2 thiourea, and were incubated for 30 min after slicing in warm (34°C), oxygenated holding solution containing the following (in millimolar (mM)): 92 NaCl, 30 NaHCO_3_, 1.2 NaH_2_PO_4_, 2.5 KCl, 35 glucose, 20 HEPES, 2 MgCl_2_, 2 CaCl_2_, 5 Na-ascorbate, 3 Na-pyruvate, and 2 thiourea. Slices were then stored at room temperature and recordings performed within 30 min to 6 h. Mice used in calcium imaging experiments were perfused transcardially with ice-cold glycerol-based solution before brain extraction and slicing.

### Electrophysiological recordings

Slices were continuously superfused at ∼2 ml/min with warm (34°C), oxygenated extracellular recording solution containing the following (in millimolar (mM)): 125 NaCl, 25 NaHCO_3_, 1.25 NaH_2_PO_4_, 3.5 KCl, 10 glucose, 1 MgCl_2_, and 2 CaCl_2_ (unless otherwise indicated). Neurons were visualized with a 40× objective using a BX51WI Olympus microscope equipped with a scientific CMOS camera (pco.edge 4.2, PCO). Cell-attached (10 MΩ to GΩ seals) and whole-cell recordings were obtained using low-resistance pipettes (3-7 MΩ) pulled from filamented borosilicate glass with a flaming/brown micropipette puller (Sutter Instruments). Cell-attached recordings performed with low seal resistance did not differ in drug response from those performed with tighter seals (*n* = 96; *r* = 0.07,225; *r*^2^ = 0.005219; *p* = 0.4842). The internal solution contained the following (in millimolar (mM)): 122 KMeSO_3_, 9 NaCl, 1.8 MgCl_2_, 4 Mg-ATP, 0.3 Na-GTP, 14 phosphocreatine, 9 HEPES, 0.45 EGTA, and 0.09 CaCl_2_.

Low calcium experiments were performed using extracellular recording solution containing the following (in millimolar (mM)): 125 NaCl, 25 NaHCO_3_, 1.25 NaH_2_PO_4_, 3.5 KCl, 10 glucose, 1 MgCl_2_, and 0.1 CaCl_2_. Calcium was lowered without adjusting the concentration of magnesium. The osmolarity of low calcium external solutions was 300-310 mOsM.

Signals were digitized with a Digidata 1590 interface, amplified by a Multiclamp 700B amplifier, and acquired using pClamp 13 software (Molecular Devices). Data were sampled at 20 kHz and filtered at 10 kHz. Data were analyzed using the NeuroMatic toolkit and custom procedures in IgorPro (WaveMetrics).

All recordings were performed in DANs. DANs were targeted by their anatomic location and presence of tdTomato or CTb-AF555, and identified based on various electrophysiological characteristics, such as the firing frequency (<5 Hz) and presence of HCN-mediated sag.

### Two-photon calcium imaging

Slices were continuously superfused with warm (34°C), oxygenated recording solution containing the following (in millimolar (mM)): 125 NaCl, 25 NaHCO_3_, 1.25 NaH_2_PO_4_, 3.5 KCl, 10 glucose, 1 MgCl_2_, and 2 CaCl_2_.

Two-photon calcium imaging was performed using a custom-built two-photon laser-scanning microscope (Sutter) operating at 940 nm (Mai Tai DeepSee; Spectra Physics) and equipped with a scientific CMOS camera (pco.edge 4.2, PCO). The fluorescent signal was detected by above-stage photomultiplier tubes (PS-2LV; Sutter). Line scans were acquired at 30 Hz with a pixel dwell time of 88 ns. Laser scanning was controlled using ScanImage software (Vidrio).

Images were analyzed offline by manually drawn ROIs and processed using custom Fiji/ImageJ macros and IgorPro (WaveMetrics) procedures. Data are presented as the relative change in fluorescence compared with baseline (ΔF/F).

### Drugs

Patch-clamp recordings and calcium imaging experiments were performed in the presence of synaptic blockers (20 μM CNQX, 50 μM APV, and 50 μM picrotoxin). As indicated, we used 100 μM gadolinium (Gd), 10 μM L-703,606, and/or 10 μM ZD-7288.

Salts were purchased from Sigma-Aldrich. Drugs were purchased from Tocris Bioscience and Sigma-Aldrich. All drugs were prepared as aliquots in water or DMSO.

### RNA fluorescence in situ hybridization (FISH)

RNAscope ISH was performed on 16-µm-thick coronal midbrain slices from fresh-frozen mouse brain cut on a cryostat (Leica CM 1950). All reagents used are commercially available from ACDBio. Procedures for the Multiplex FISH assay were followed as recommended by ACDBio.

Slices were imaged on a Zeiss LSM 800 confocal microscope using Zen Blue software in the National Institute of Neurological Disorders and Stroke Light Imaging Core Facility. Images were analyzed by manually drawn ROIs and processed using a custom Fiji/ImageJ macro.

### Data analysis

Data were analyzed using Prism (GraphPad software) and IgorPro (Wavemetrics). Statistical significance in two group comparisons was determined using Mann–Whitney *U* tests (unpaired), Wilcoxon signed-rank tests (paired), or one-way ANOVAs (multiple groups). Data are reported as mean ± SEM. Nonparametric tests were used because of small sample sizes. Averages were calculated using the last 10 sweeps of the drug wash-in.

## Results

### NALCN RNA expression varies across dopaminergic neuron subpopulations

**Figure 1.**
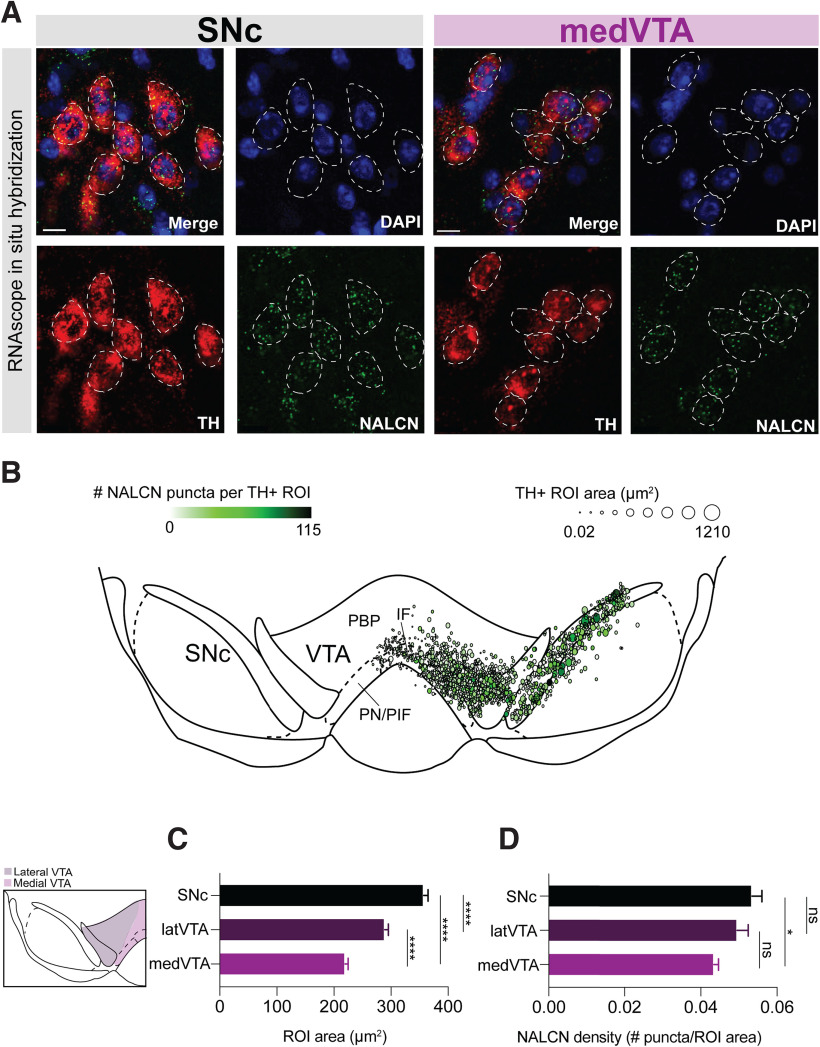
NALCN RNA expression across midbrain dopaminergic neurons. ***A***, ISH on midbrain coronal sections. Confocal image of TH-expressing neurons (red) and NALCN-positive neurons (green) in the SNc and VTA. Scale bar, 10 μm. ***B***, Expression map of NALCN RNA expression. Marker color represents the number of NALCN puncta in each ROI. Marker size indicates the area of each ROI. Expression maps from four slices are overlaid. ***C***, Average TH defined ROI area for cells in SNc, latVTA, and medVTA (*p* < 0.0001). ***D***, NALCN expression density in SNc (*n* = 448), latVTA (*n* = 469; *p* = 0.18), and medVTA (*n* = 517; *p* = 0.03). *p < 0.05. ****p < 0.0001.

To determine the RNA expression of the sodium leak channel, NALCN, in subpopulations of DANs located in the SNc, lateral VTA (latVTA), and ventromedial VTA (medVTA), we performed in situ hybridization of RNA from NALCN on fixed midbrain tissue slices from adult mice (Fig. 1*A*,*B*). DANs were identified according to the presence of RNA for tyrosine hydroxylase (TH), which was also used to define the region of interests (ROIs) surrounding individual soma of DANs. Comparing the size of neurons as defined by the area of their ROIs, we found that DANs in both the medVTA (219.5 ± 5.24 µm^2^; *n* = 517) and the latVTA (289.1 ± 6.2 µm^2^; *n* = 469) were significantly smaller than those in the SNc (357.3 ± 7.71 µm^2^; *n* = 448) (Fig. 1*C*; medVTA vs SNc, *p* < 0.0001; latVTA vs SNc; *p* < 0.0001; medVTA vs latVTA, *p* < 0.0001; four slices).

We next quantified the density of NALCN particles among DAN subpopulations by normalizing to the ROI area for each neuron (density = number of NALCN puncta/ROI area). We found that the density of NALCN expression in the latVTA DANs was not significantly different from that of the SNc (Fig. 1*D*; *p* = 0.18). However, medVTA DANs had significantly lower expression density compared with the SNc (Fig. 1*D*; *p* = 0.03). Therefore, NALCN RNA is present in all DANs, with slightly weaker expression in medVTA relative to SNc DANs.

### Pharmacological inhibition of NALCN slows pacemaker firing in medial and lateral VTA dopaminergic neuron subpopulations

Past work has established that ionic conductances that support firing can differ substantially in VTA DANs relative to “canonical” SNc neurons. Specifically, VTA DANs exhibit smaller voltage sags, indicating lower levels of HCN channels (Neuhoff et al., 2002; Margolis et al., 2006; Lammel et al., 2008; Kramer et al., 2018). Additionally, in experiments in which extracellular sodium is replaced with *N*-methyl-D-glucamine, VTA neurons exhibit a larger subthreshold sodium leak current (Khaliq and Bean, 2010). However, the functional role of NALCN in pacemaking of VTA DANs has not been previously examined.

**Figure 2.**
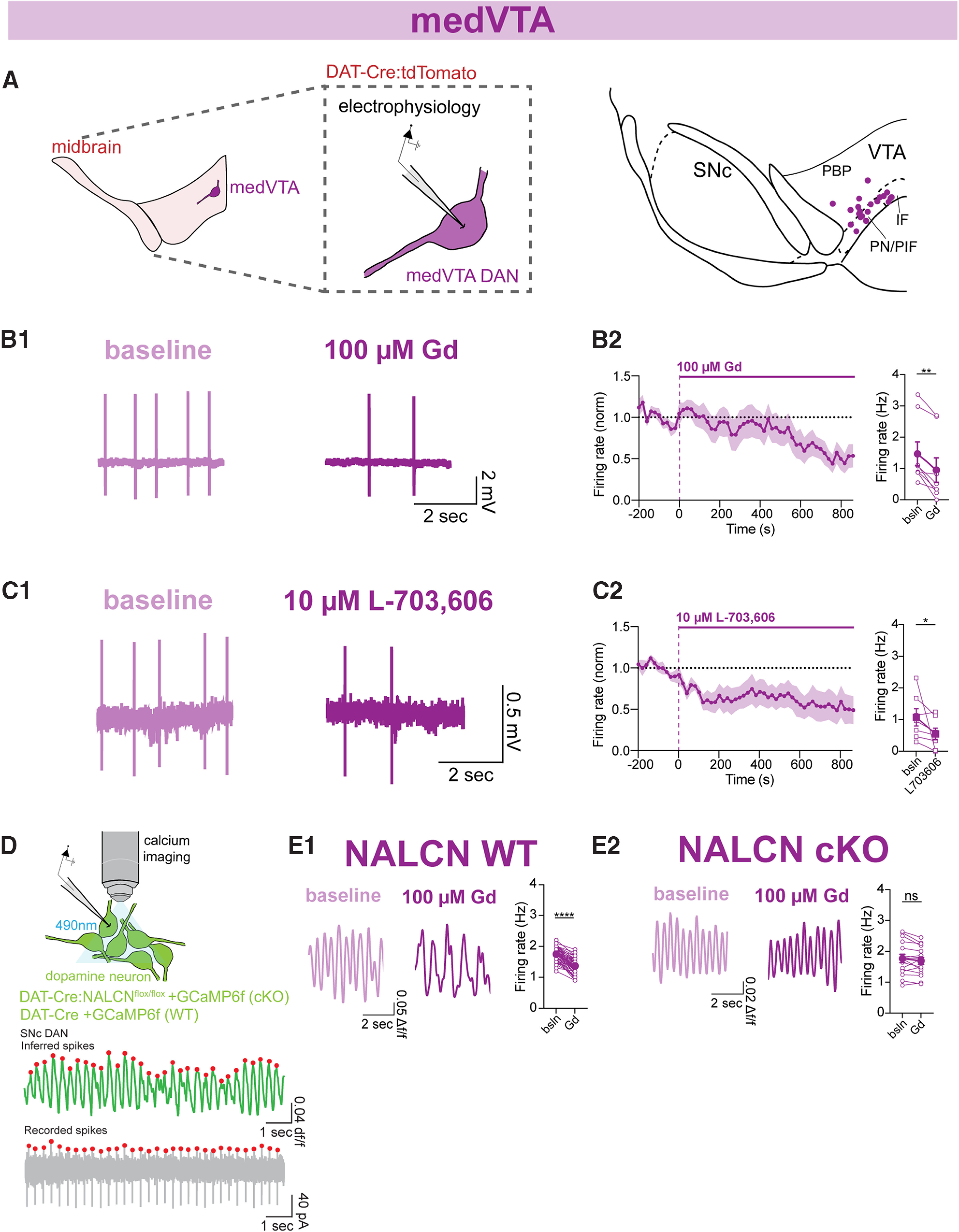
Inhibition of NALCN by Gd or L-703,606 slows pacemaking in medVTA dopaminergic neurons from WT but not NALCN cKO mice. ***A***, Cell-attached electrophysiology was performed on anatomically defined medVTA DANs using fluorescently labeled cells in DAT-Cre::tdTomato mice. Map of recording locations in VTA. ***B1***, Example trace of cell-attached recordings of firing rate before (light purple) and after (dark purple) Gd in medVTA DANs. ***B2***, Left, Timecourse of 100 μM Gd (14 min) effect on the firing rate of medVTA DANs recorded in cell-attached mode. Right, Averaged firing rate of medVTA DANs before and after Gd (*n* = 8; *p* = 0.008). ***C1***, Example trace of cell-attached recordings of firing rate before (light purple) and after (dark purple) L-703,606 in medVTA DANs. ***C2***, Left, Timecourse of 10 μM L-703,606 (14 min) effect on the firing rate of medVTA DANs recorded in cell-attached mode. Right, Averaged firing rate of medVTA DANs before and after L-703,606 (*n* = 7; *p* = 0.03). ***D***, Simultaneous cell-attached electrophysiology and calcium imaging were performed on DANs. Example traces of simultaneous calcium imaging (top) and cell-attached electrophysiology (bottom) from a single cell. ***E1***, Left, Example trace of spontaneous calcium imaging signal from a single NALCN WT medVTA DAN before (light purple) and after (dark purple) Gd. Right, Averaged firing rate as measured with calcium imaging of NALCN WT medVTA DANs before and after 100 μM Gd (*n* = 51, *p* < 0.0001). ***E2***, Left, Example trace of spontaneous calcium imaging signal from a single NALCN cKO medVTA DAN before (light purple) and after (dark purple) Gd. Right, Averaged firing rate as measured with calcium imaging of NALCN cKO medVTA DANs before and after 100 μM Gd (*n* = 16; *p* = 0.07). *p < 0.05. **p < 0.01. ****p < 0.0001.

We tested the functional contribution of NALCN to cell-attached pacemaking of DANs located in the ventromedial region of the VTA (Fig. 2*A*, medVTA) by applying pharmacological blockers of NALCN to the bath solution. We first tested gadolinium (Gd), a nonspecific blocker of NALCN (Lu et al., 2007; Hahn et al., 2020). Bath application of Gd resulted in 36.05% reduction in the spontaneous firing rate, from 1.47 ± 0.38 to 0.94 ± 0.38 Hz (Fig. 2*B*; *n* = 8, *p* = 0.008). We next tested a separate small molecule drug, L-703,606, which has been shown to be a specific inhibitor for NALCN at concentrations up to 10 μM (Hahn et al., 2020). Acute blockade of NALCN with 10 μM L-703,606 in medVTA DANs reduced spontaneous firing rate by 48.59%, from 1.07 ± 0.27 to 0.55 ± 0.19 Hz (Fig. 2*C*; *n* = 7; *p* = 0.03). Comparison of the effect on firing when using Gd (*n* = 8) or L-703,606 (*n* = 7) showed no significant difference in the reduction of firing rate caused by either of the two drugs (*p* = 0.89). Last, we found that Gd had little to no effect on the half-width of action potentials in medVTA DANs (half-width: control, 2.03 ± 0.23 ms; Gd, 2.15 ± 0.33 ms; *n* = 9; *p* = 0.49).

As an alternative to electrophysiological recordings, we used two-photon calcium imaging to monitor single action potential-evoked calcium signals in medVTA DANs. Calcium signals were monitored using GCaMP6f that was expressed in DANs using adeno-associated viruses injected into DAT-Cre mice. Simultaneous cell-attached electrical recording of pacemaking and two-photon calcium imaging confirmed that GCaMP6f signals could be used to accurately identify action potential evoked-calcium signals produced during single action potentials (Fig. 2*D*). Consistent with our electrophysiology results, we found that the inferred pacemaker firing rate of medVTA DANs from calcium imaging was significantly slowed following acute blockade of NALCN with Gd (Fig. 2*E1*; *n* = 51, *p* < 0.0001). Additionally, we found that the normalized averaged calcium signal, a read-out of intracellular calcium concentration, was reduced to a similar extent (*p* = 0.1) under control conditions (*n* = 30) and in the presence of Gd (*n* = 132) after 14 min of imaging, likely because of normal rundown or bleaching of the GCaMP6f indicator.

To determine whether Gd slowed firing through nonspecific interactions with targets other than NALCN, we tested the effect of Gd on midbrain slices from NALCN cKO mice in which NALCN was deleted in DANs. Consistent with past work (Philippart and Khaliq, 2018), we found that the majority of DANs are silent in the NALCN cKO mice while only a subset of cells were spontaneously active. Importantly, when we tested firing in the subset of spontaneously active medVTA DANs from NALCN cKO mice using calcium imaging, we found that their pacemaker rate was unaffected by the application of Gd (Fig. 2*E2*; *n* = 16 cells; *p* = 0.07). These data demonstrate that Gd slows pacemaking in medVTA DANs mainly through inhibition of NALCN rather than nonspecific effects. Together, these results identify NALCN as the main molecular correlate of the sodium leak conductance in medVTA DANs and a prominent driver of pacemaking in these cells.

**Figure 3.**
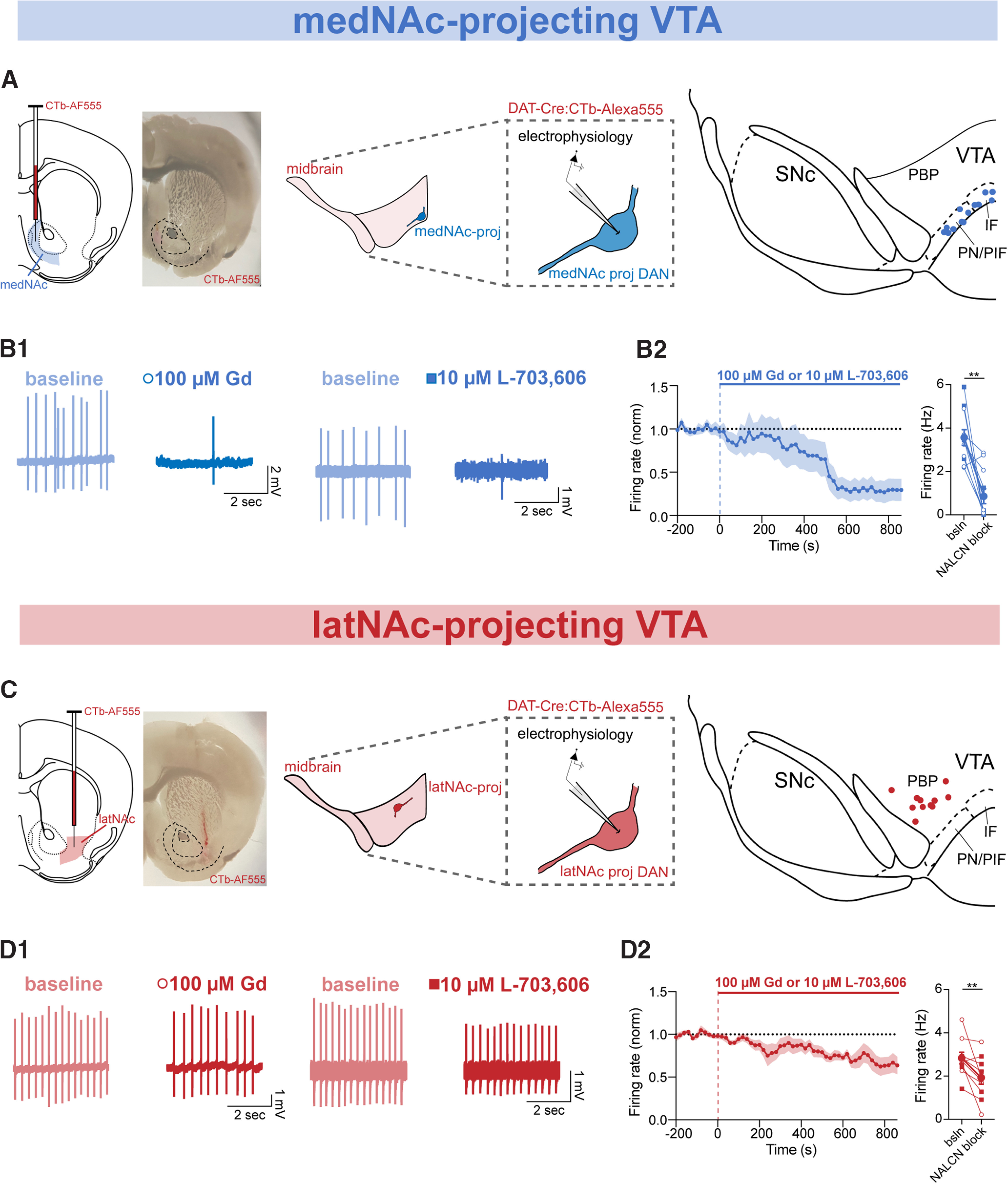
Inhibition of NALCN with Gd or L-703,606 slows pacemaking in medNAc-projecting and latNAc-projecting VTA dopaminergic neurons. ***A***, Left, CTb-Alexa555 was injected bilaterally into the medNAc. Right, Cell-attached electrophysiology was performed on retrogradely labeled medNAc-projecting DANs. Map of recording locations. ***B1***, Example trace of cell-attached recordings of firing rate before (light blue) and after (dark blue) Gd (left) or L-703,606 (right) in medNAc-projecting VTA DANs. ***B2***, Left, Timecourse of 100 μM Gd or 10 μM L-703,606 (14 min) effect on the firing rate of medNAc-projecting VTA DANs recorded in cell-attached mode. ***B2***, Right, Averaged firing rate of medNAc-projecting VTA DANs before and after Gd (open circle) and/or L-703,606 (closed square) (*n* = 11, *p* = 0.002). ***C***, Left, CTb-Alexa555 was injected bilaterally into the latNAc. Right, Cell-attached electrophysiology was performed on retrogradely labeled latNAc-projecting DANs. Map of recording locations in VTA. ***D1***, Example trace of cell-attached recordings of firing rate before (light red) and after (dark red) Gd (left) or L-703,606 (right) in latNAc-projecting VTA DANs. ***D2***, Left, Timecourse of 100 μM Gd or 10 μM L-703,606 (14 min) effect on the firing rate of latNAc-projecting VTA DANs recorded in cell-attached mode. Right, Averaged firing rate of latNAc-projecting VTA DANs before and after Gd (open circle) and/or L-703,606 (closed square) (*n* = 10; *p* = 0.006). **p < 0.01.

Distinct molecular and physiological properties and behavioral functions of DANs are associated with the target structures to which they project (Lammel et al., 2008; Anderegg et al., 2015; Kramer et al., 2018; Poulin et al., 2018). Therefore, we tested the effect of acute blockade of NALCN with Gd or L-703,606 on pacemaking in projection-defined VTA DAN subpopulations. We first labeled VTA DANs that project to the medNAc by injecting cholera toxin subunit B conjugated to Alexa Fluor 555 (CTb) into the medNAc. The cell bodies of these neurons were located primarily in the ventromedial VTA (Fig. 3*A*). Bath application of either Gd (circle) or L-703,606 (square) slowed spontaneous firing in medNAc-projecting VTA DANs by 70.74%, from 3.59 ± 0.37 to 0.86 ± 0.35 Hz (Fig. 3*B*; *n* = 11, *p* = 0.002). We found no difference in effect on firing when using Gd vs L-703,606 in medNAc-projecting VTA DANs (Gd: *n* = 6, L-703,606: *n* = 5; *p* = 0.17).

We next tested retrogradely labeled VTA DANs projecting to the lateral NAc (latNAc). The cell bodies of these neurons were located primarily in the dorsolateral VTA (Fig. 3*C*). Acute blockade of NALCN with Gd (circle) or L-703,606 (square) had a much weaker effect on latNAc-projecting DAN firing, resulting in a 31.98% slowing of the spontaneous firing, from 2.81 ± 0.27 to 1.9 ± 0.31 Hz (Fig. 3*D*; *n* = 10, *p* = 0.006). There was no difference in effect on firing when using Gd or L-703,606 in latNAc-projecting VTA DANs (Gd: *n* = 5, L-703,606: *n* = 5; *p* = 0.42) cells. Together, these data demonstrate that all VTA DANs rely on NALCN for pacemaking, with medNAc-projecting DANs relying more heavily on NALCN for pacemaking relative to those projecting to the lNAc.

### Inhibition of NALCN with either Gd or L-703,606 does not affect pacemaking in SNc dopaminergic neurons

**Figure 4.**
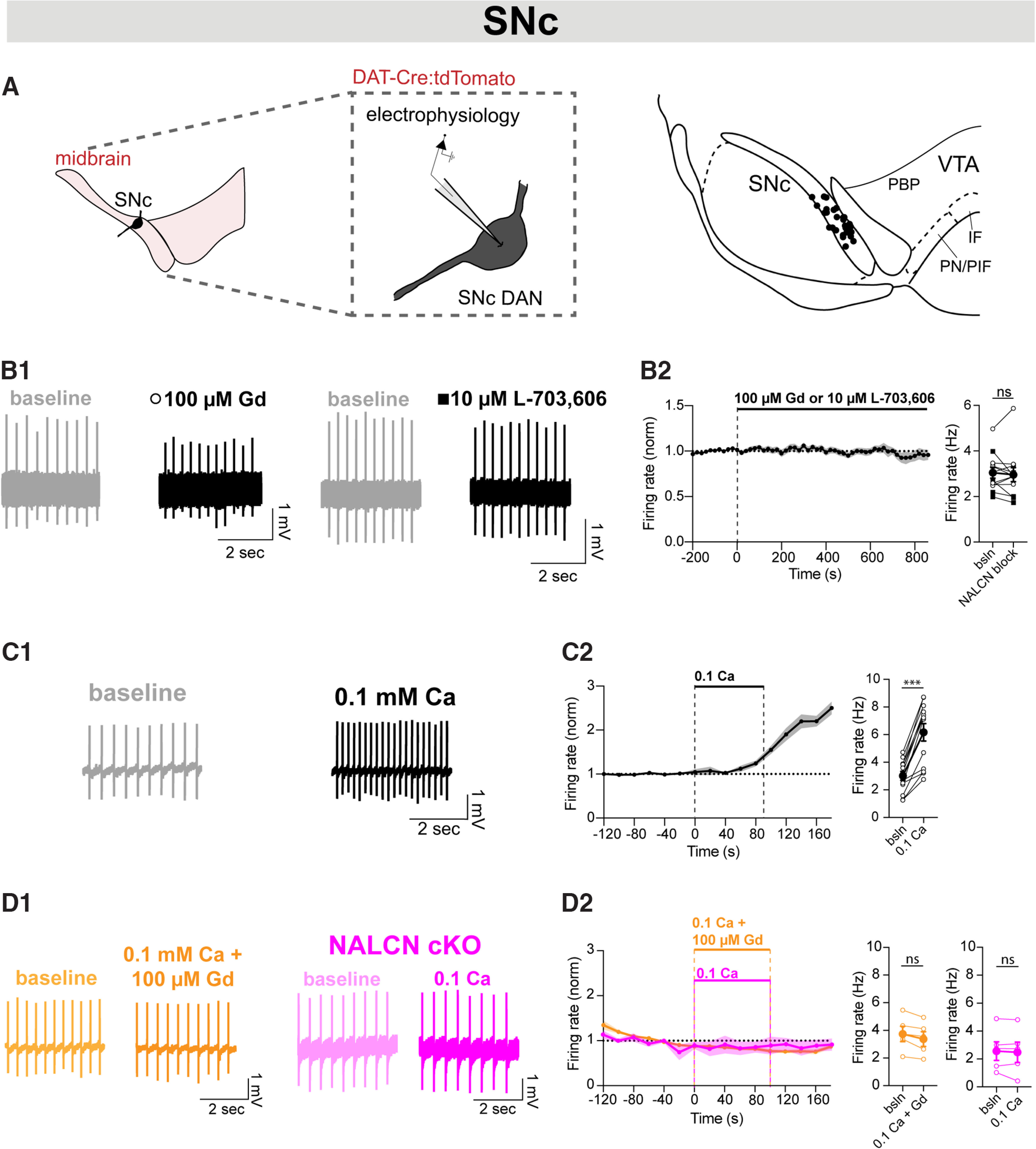
Inhibition of NALCN with Gd or L-703,606 does not affect pacemaking in SNc dopaminergic neurons. ***A***, Cell-attached electrophysiology was performed on anatomically defined SNc DANs using tdTomato fluorescence. Map of recording locations in SNc. ***B1***, Example trace of cell-attached recordings of firing rate before (gray) and after (black) Gd (left) or L-703,606 (right) in SNc DANs. ***B2***, Left, Timecourse of firing rate following 100 μM Gd or 10 μM L-703,606 (14 min) applied to cells recorded in cell-attached mode. Right, Averaged firing rate before and after Gd (open circle) and/or L-703,606 (closed square) (*n* = 12; *p* = 0.68). ***C1***, Example trace of cell-attached recordings of firing rate before (gray) and after (black) 90 s of low calcium in SNc DANs. ***C2***, Left, Timecourse of firing rate following low external calcium (0.1 mm, 90 s) applied to SNc DANs. Right, Averaged firing rate of SNc DANs before and after low calcium (*n* = 13; *p* = 0.0002). ***D1***, Left, Example trace of cell-attached recordings of firing rate before (light orange) and after (dark orange) 0.1 mM calcium and 100 μM Gd in SNc DANs. Right, Example trace of cell-attached recordings of firing rate before (light pink) and after (dark pink) 90 s of low calcium in NALCN cKO SNc DANs. ***D2***, Left, Timecourse of firing rate following concurrent low external calcium and Gd (0.1 mM Ca, 100 μM Gd, 90 s, orange) or low external calcium applied to spontaneously active NALCN cKO cells (0.1 mM Ca, 90 s, pink). Right, Averaged firing rate before and after low calcium and in Gd (*n* = 5; *p* = 0.06; orange). Averaged firing rate of NALCN cKO SNc DANs before and after low calcium (*n* = 5; *p* = 0.56; pink). ***p < 0.001.

Studies examining the role of NALCN in pacemaking of SNc DANs have been conflicting, with some studies showing a dominant role of NALCN to pacemaking in SNc and others showing little to no contribution. Therefore, we investigated the contribution of NALCN to pacemaking of medial SNc DANs (Fig. 4*A*). We found that acute blockade of NALCN with Gd (circle) or L-703,606 (square) had little effect (3.03% inhibition) on the firing rate of medial SNc DANs (Fig. 4*B*; *n* = 12, *p* = 0.68), in agreement with previous studies showing little to no contribution of NALCN to pacemaking of NALCN (Jehasse et al., 2021). Interestingly in whole-cell mode, we found that Gd led to broader action potentials (baseline: 1.92 ± 0.15 ms, Gd: 2.49 ± 0.23 ms; *n* = 6; *p* = 0.03) and shorter spike heights (baseline: 85.62 ± 2.79 mV, Gd: 73.42 ± 3.75 mV; *n* = 6; *p* = 0.03), suggesting nonspecific effects of Gd on spike-evoked currents. Additionally, the average interspike membrane potential was largely unaffected in SNc DANs (pre: −54.23 ± 1.77 mV, post: −54.80 ± 1.54 mV; *n* = 6; *p* = 0.53), and there was no difference in effect when using Gd (*n* = 7) or L-703,606 (*n* = 7; *p* = 0.26). We repeated this set of experiments using two-photon calcium imaging of activity in SNc DA neurons and found a small but significant increase in the inferred spike rates from evoked calcium signals from 1.57 ± 0.03 to 1.76 ± 0.03 Hz, likely detectable because of the large sample size (*n* = 124; *p* < 0.0001). Together, these results suggest that the lack of effect on SNc DANs firing is unlikely to result primarily from complex nonspecific effects of Gd.

Our ISH results (Fig. 1) suggested that NALCN is expressed at high levels in SNc DANs; therefore, we wanted to test the functional expression of NALCN in the SNc by potentiating the NALCN current. It is well established that lowering extracellular calcium concentrations below physiological levels of ∼1.2-2 mM increases neuronal excitability (Frankenhaeuser and Hodgkin, 1957; Lu et al., 2010; Jones and Smith, 2016; Philippart and Khaliq, 2018; Forsberg et al., 2019; Chua et al., 2020). Additionally, previous work has shown that extracellular calcium impedes the flow of sodium ions through the main pore of the NALCN channel (Chua et al., 2020; Kang et al., 2020; Kang and Chen, 2022), and that lowering extracellular calcium potentiates the NALCN current and increases firing in HEK cells (Lu et al., 2009; Chua et al., 2020) and DANs (Philippart and Khaliq, 2018).

Therefore, as a functional assay for the presence of NALCN, we tested a 90 s wash-in of low extracellular (0.1 mM) calcium to potentiate the NALCN current. We found that low extracellular calcium significantly increased firing of SNc DANs from 2.97 ± 0.32 to 6.14 ± 0.63 Hz (Fig. 4*C*; *n* = 13; *p* = 0.0002). This increase was blocked by the addition of Gd (Fig. 4*D*; orange; *n* = 5; *p* = 0.06) and was absent in NALCN cKO cells that were firing spontaneously (Fig. 4*D*; pink; *n* = 5; *p* = 0.56). Additionally, NALCN cKO cells that were silent at baseline could not be induced to fire in low extracellular calcium (control: 0 Hz; low Ca: 0 Hz; *n* = 10). Together, these data suggest that, although NALCN is not a primary driver of pacemaker firing in SNc DANs, NALCN is functionally expressed in these cells.

### NALCN inhibition alters the gain of the frequency-current curve for firing rates slower than pacemaker frequencies in SNc dopaminergic neurons

**Figure 5.**
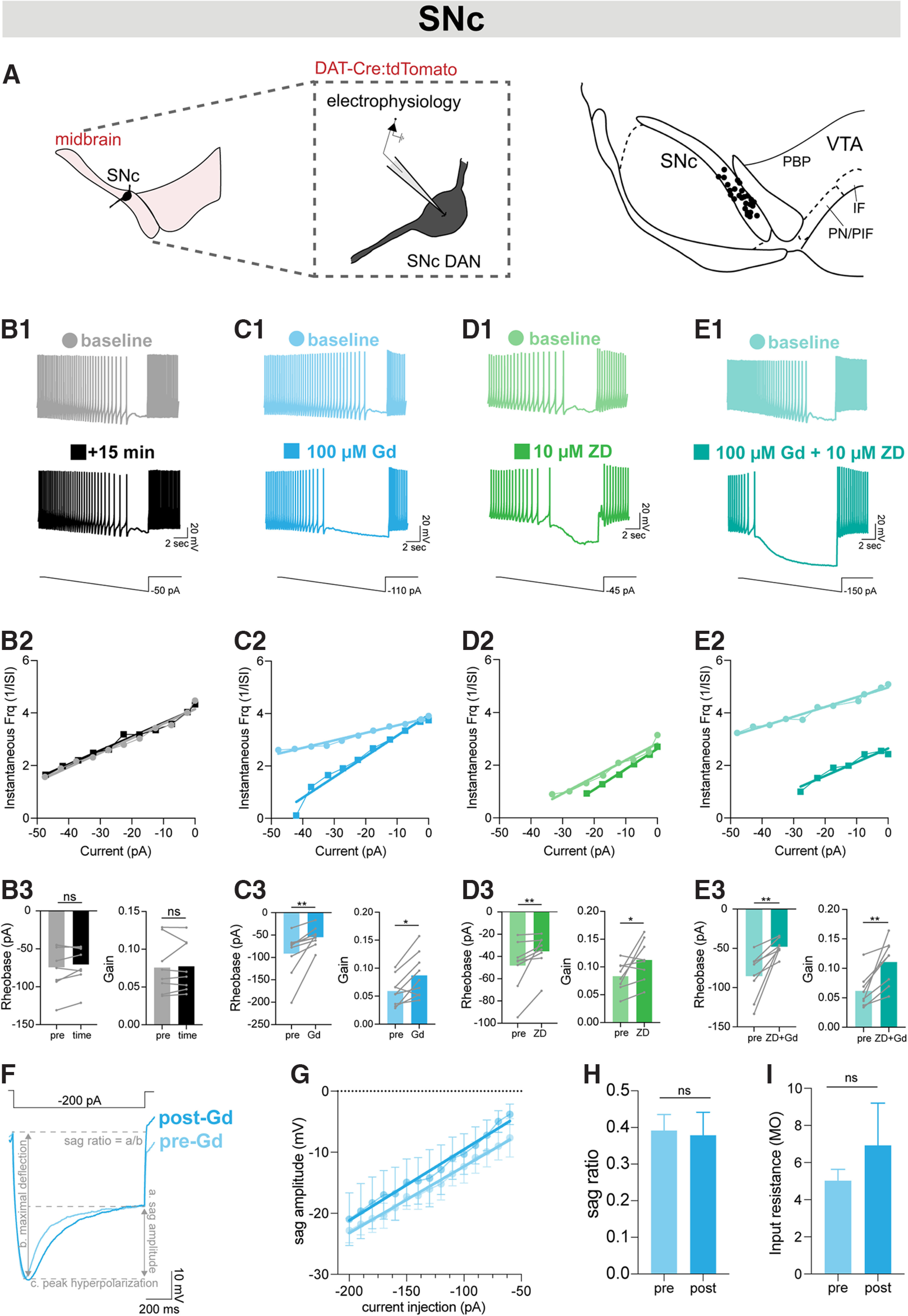
NALCN inhibition alters the gain of the frequency-current curve for firing rates slower than pacemaker frequencies in SNc dopaminergic neurons. ***A***, Whole-cell electrophysiology was performed on anatomically defined SNc DANs using tdTomato fluorescence. Map of recording locations in SNc. ***B1***, Example trace of whole-cell recordings of action potentials during 15 s hyperpolarizing ramp at baseline (light gray) and after 15 min time control (black). ***B2***, Paired instantaneous frequency plotted against current injection with time control before (gray) and after (black) 15 min. ***B3***, Effect of time on gain (baseline: 0.076 ± 0.013 Hz/pA, time control: 0.077 ± 0.012 Hz/pA, *p* = 0.74, *n* = 8) and rheobase (baseline: −74.32 ± 10.63 pA, time control: −70.62 ± 8.92 pA, *p* = 0.25, *n* = 8). ***C1***, Example trace of whole-cell recordings of action potentials during 15 s hyperpolarizing ramp at baseline (light blue) and after 15 min 100 μM Gd (dark blue). ***C2***, Paired instantaneous frequency plotted against current injection before (light blue) and after (dark blue) 100 μM Gd. ***C3***, Effect of Gd on rheobase (baseline: −91.61 ± 16.33 pA, Gd: −54.58 ± 7.28 pA; *p* = 0.004, *n* = 9) and gain (baseline: 0.059 ± 0.009 Hz/pA, Gd: 0.087 ± 0.013 Hz/pA; *p* = 0.02, *n* = 9). ***D1***, Example trace of whole-cell recordings of action potentials during 15 s hyperpolarizing ramp at baseline (light green) and after (dark green) 15 min 10 μM ZD. ***D2***, Paired instantaneous frequency plotted against current injection before (light green) and after (dark green) 10 μM ZD. ***D3***, Effect of ZD on rheobase (baseline: −48.19 ± 8.21 pA, ZD-7288: −35.36 ± 5.65 pA; *p* = 0.008, *n* = 8) and gain (baseline: 0.083 ± 0.009 Hz/pA, ZD-7288: 0.113 ± 0.013 Hz/pA; *p* = 0.04, *n* = 8), although these changes were slightly smaller than those seen with Gd (***D1-D3***). ***E1***, Example trace of whole-cell recordings of action potentials during 15 s hyperpolarizing ramp at baseline (light teal) and after (dark teal) 15 min 100 μM Gd + 10 μM ZD. ***E2***, Paired instantaneous frequency plotted against current injection before (light teal) and after (dark teal) 100 μM Gd + 10 μM ZD. ***E3***, Effect of ZD+Gd on rheobase (baseline: −85.40 ± 10.27 pA; Gd+ZD: −47.90 ± 3.64 pA; *p* = 0.008, *n* = 8) and gain (baseline: 0.0612 ± 0.01 Hz/pA, post: 0.111 ± 0.14 Hz/pA; *p* = 0.0078, *n* = 8). ***F***, Paired example traces of whole-cell current-clamp recordings during hyperpolarizing steps to evoke HCN currents before (black) and after (teal) Gd. ***G***, Sag amplitude (mV) plotted versus current injection (pA) from −60 to −200 mV before (black) and after (teal) Gd. ***H***, Sag ratio of paired cells at −200 pA hyperpolarizing steps before (black) and after (teal) Gd (*n* = 5, *p* = 0.38). ***I***, Quantified input resistance (slope) before (black) and after (teal) Gd (*n* = 5, *p* > 0.99). *p < 0.05. **p < 0.01.

Although we found that NALCN does not drive pacemaking in SNc DANs, we wondered whether NALCN supports firing at frequencies below pacemaker rates. To test this, we performed whole-cell recordings of SNc DANs and applied long-duration (15 s) hyperpolarizing current ramps (Fig. 5*A*). For each cell, the maximal ramp current injection was individually tuned (−25 to −165 pA) to inhibit action potential generation in the last 3 s of the injection. The instantaneous frequency of each cell was plotted against the current injection at a given time point to generate a frequency-current (f-I) curve. A simple linear regression was performed to determine the gain (defined as the slope) and rheobase (defined as the *x*-intercept) for each cell.

There was no effect on the gain (baseline: 0.076 ± 0.01 Hz/pA, time control: 0.077 ± 0.01 Hz/pA, *p* = 0.7422, *n* = 8) or rheobase (baseline: −74.32 ± 10.63 pA, time control: −70.62 ± 8.92 pA, *p* = 0.2500, *n* = 8) in the time control condition, as expected (Fig. 5*B*). By contrast, we found that acute blockade of NALCN with Gd shifted the rheobase to be less negative (baseline: −91.61 ± 16.33 pA, Gd: −54.58 ± 7.28 pA; *p* = 0.004, *n* = 9) and increased the gain (baseline: 0.059 ± 0.009 Hz/pA, Gd: 0.087 ± 0.013 Hz/pA; *p* = 0.02, *n* = 9; Fig. 5*C*). This suggests that NALCN supports firing in SNc DANs at frequencies below pacemaker rates.

Because hyperpolarization also recruits HCN channels which are highly expressed in SNc DANs (Mercuri et al., 1995; Neuhoff et al., 2002), we wanted to examine the contribution of HCN channels to this observation. We observed that acute blockade of HCN channels with 10 μM ZD-7288 also resulted in a positive shift in the rheobase (baseline: −48.19 ± 8.21 pA, ZD-7288: −35.36 ± 5.65 pA; *p* = 0.008, *n* = 8) and the gain (baseline: 0.083 ± 0.009 Hz/pA, ZD-7288: 0.113 ± 0.013 Hz/pA; *p* = 0.04, *n* = 8), although these changes were slightly smaller than those seen with Gd (Fig. 5*D*). We found that acute blockade of both NALCN and HCN channels also shifted the rheobase (baseline: −85.40 ± 10.27 pA; Gd+ZD: −47.90 ± 3.64 pA; *p* = 0.008, *n* = 8) and magnified the effect on the gain (baseline: 0.0612 ± 0.01 Hz/pA, post: 0.111 ± 0.14 Hz/pA; *p* = 0.008, *n* = 8; Fig. 5*E*). These results are independent of blockade of HCN channels, as Gd had little effect on the sag voltage (Fig. 5*F–I*; *n* = 5; *p* = 0.3750) and input resistance of the cell (Fig. 5*I*; *n* = 5; *p* > 0.99).

**Figure 6.**
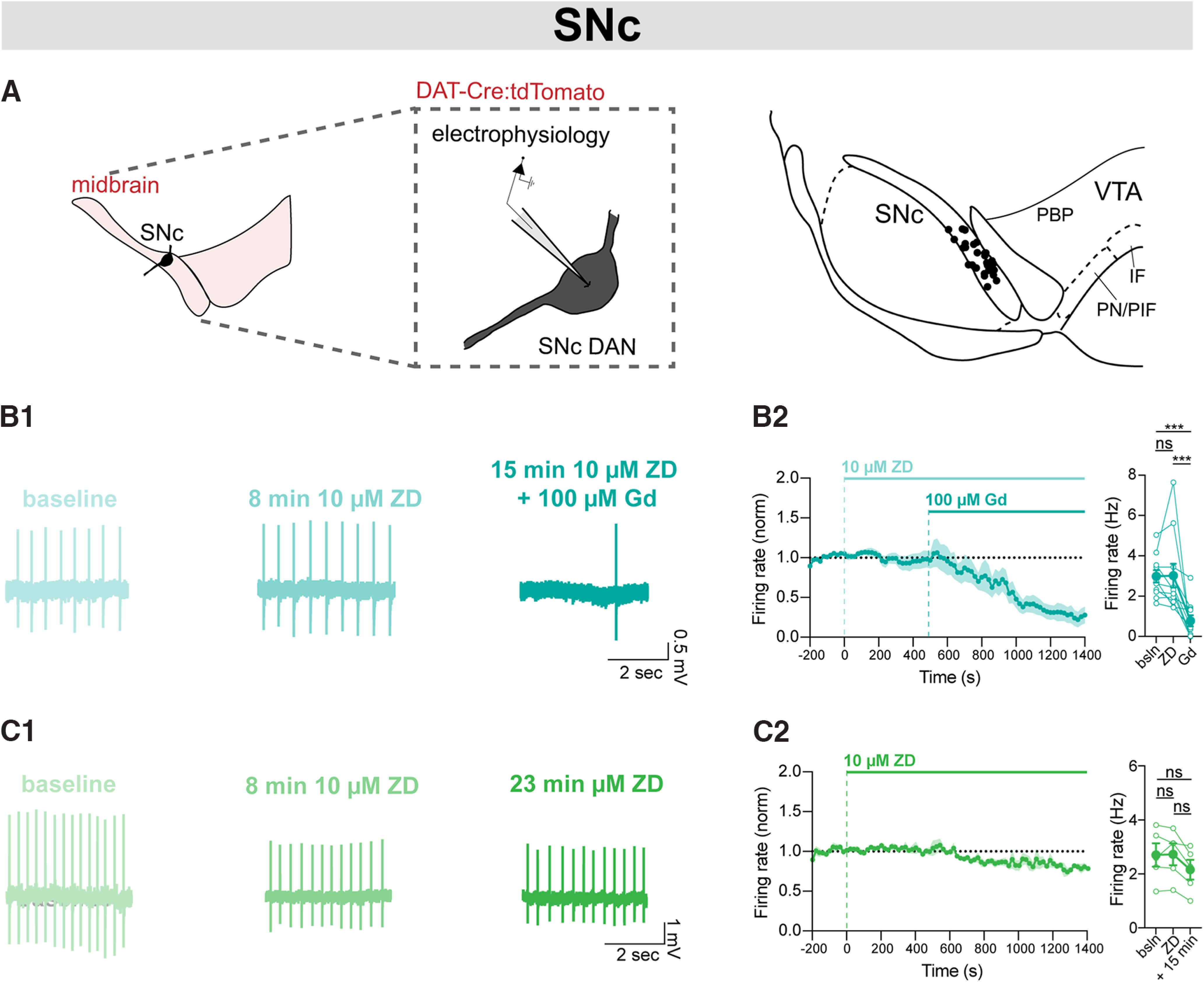
Simultaneous blockade of NALCN and HCN channels inhibits pacemaking in SNc DANs. ***A***, Cell-attached electrophysiology was performed on anatomically defined SNc DANs using tdTomato fluorescence. Map of recording locations in SNc. ***B1***, Example trace of cell-attached recordings of firing rate at baseline (light teal), after ZD-7288 (teal), and after ZD and Gd (dark teal). ***B2***, Left, Timecourse of firing rate following application of ZD (10 μM, 8 min) and Gd (100 μM, 15 min) onto cells recorded in cell-attached mode. Right, Averaged firing rate at baseline, after ZD-7288 (*n* = 11; *p* = 0.93), and after ZD-7288 plus Gd (*n* = 11; *p* = 0.0003). ***C1***, Example trace of cell-attached recordings of firing rate at baseline (light green), after 8 min ZD-7288 (dark green), and after 23 min ZD-7288 (dark green). ***C2***, Left, Timecourse of firing rate following 8 min ZD-7288 (10 μM) and 23 min ZD-7288 application onto cells recorded in cell-attached mode. Right, Averaged firing rate at baseline, after 8 min ZD-7288 (*n* = 5; *p* = 0.88), and after 23 min ZD-7288 (*n* = 5; *p* = 0.06). ***p < 0.001.

To further explore this compounded effect on the excitability of SNc DANs under acute blockade of both NALCN and HCN channels, we performed cell-attached recordings and measured the effect on spontaneous firing in SNc DANs (Fig. 6*A*). Consistent with our results in Figure 5*E*, we found that acute blockade of NALCN and HCN channels with 100 μM Gd and 10 μM ZD-7288 resulted in robust inhibition of pacemaking in SNc DANs (Fig. 6*B*; *n* = 11; *p* = 0.001). These effects were not because of Gd blocking HCN channels, as acute blockade of HCN channels alone had no effect on firing (Fig. 6*C*; *n* = 5; *p* = 0.06). Together, this demonstrates that simultaneous block of both NALCN and HCN inhibits the firing of SNc DANs, suggesting that, when firing is slowed, removal of each conductance has a more substantial effect on firing.

## Discussion

We investigated the contribution of the sodium leak channel, NALCN, to pacemaking in DAN subpopulations. We found that NALCN is a dominant contributor to pacemaking of VTA DANs. Specifically, medNAc-projecting VTA DANs display the largest reliance on NALCN for pacemaking and latNAc-projecting VTA DANs display slightly less reliance on NALCN. By contrast, we found that blockade of NALCN has no effect on pacemaking in SNc DANs. Rather, NALCN helps set the gain of the frequency-current curve for firing rates that are below pacemaker frequencies in SNc DANs. These findings expand our understanding of how pacemaking occurs in DAN subpopulations, adding to a body of literature demonstrating that SNc and VTA DANs rely on different pacemaking mechanisms.

### Heterogeneity in contribution of NALCN to pacemaking of midbrain dopaminergic neurons

Pacemaking of DANs depends on multiple subthreshold conductances active during the interspike interval (Puopolo et al., 2007; Khaliq and Bean, 2010; Gantz et al., 2018). This includes voltage-dependent conductances, such as HCN channels, L-type calcium channels, persistent sodium currents (Mercuri et al., 1995; Crill, 1996; Puopolo et al., 2007), and voltage-independent conductances, such as sodium leak (Khaliq and Bean, 2010; Um et al., 2021). In this study, we precisely defined the role of NALCN in pacemaking of DAN subpopulations. We show that pacemaking in VTA DANs is reduced by acute blockade of NALCN, with VTA DANs projecting to the medNAc showing the largest reduction in firing and VTA DANs projecting to the latNAc showing a smaller reduction in firing. This is consistent with past studies showing that medVTA neurons express low levels of HCN channels (Margolis et al., 2006; Lammel et al., 2008, 2014; Kramer et al., 2018; Baik, 2020) and increased reliance on a voltage-independent sodium leak conductance (Khaliq and Bean, 2010). Importantly, we demonstrate that NALCN is the molecular correlate of at least part of this conductance in VTA DANs and that VTA DANs rely heavily on NALCN during pacemaking.

By contrast, we found that NALCN is not critical for driving pacemaking in SNc DANs. Rather, NALCN contributes to firing at frequencies lower than typical pacemaker firing rates. In support of this idea, pharmacological inhibition of NALCN increases the gain of frequency-current plots for slow firing rates and makes the rheobase value less negative. Thus, the amplitude of negative current required to silence a pacemaking cell is lower when NALCN is inhibited. Recent work demonstrated a role of constitutive NALCN activity in proximal dendrites of SNc DANs in lowering the threshold for burst firing (Hahn et al., 2023). Combined with our data, this suggests that the presence of NALCN on SNc DANs renders these cells more excitable — more likely to burst in response to depolarizing inputs and less likely to pause firing in response to hyperpolarizing inputs.

Additionally, we found that HCN channels do not drive pacemaking in SNc DANs, but that HCN contributes to firing at slow frequencies. Importantly, we found that simultaneous inhibition of both NALCN and HCN inhibits pacemaking in SNc DANs, suggesting that, combined, NALCN and HCN make an important contribution to pacemaking in the SNc. These data are consistent with previous work showing no contribution of NALCN or HCN channels to pacemaking of SNc DANs (Mercuri et al., 1995; Seutin et al., 2001; Neuhoff et al., 2002; Khaliq and Bean, 2010; Jehasse et al., 2021) but adds to existing knowledge of how these highly expressed ion channels contribute to firing of SNc DANs.

Combined, these results suggest that NALCN contributes differentially to pacemaking and excitability of DAN subpopulations. We demonstrated that, although NALCN does not directly contribute to pacemaking of SNc DANs, it plays an important role in regulating excitability of these cells. By contrast, we identified NALCN as a dominant contributor to pacemaking of VTA DANs projecting to the medNAc, while NALCN plays a less robust role in VTA DANs projecting to the latNAc. Thus, future studies examining the projection-specific and subpopulation-specific differences in regulation of DAN excitability should include NALCN.

### Contribution of sodium leak conductances to pacemaking of midbrain dopaminergic neurons

Our data identify NALCN as the molecular correlate of at least part of the sodium leak conductance that provides subthreshold depolarization to drive pacemaking of DANs. However, the identity of all molecular components that contribute to sodium leak conductance in DANs is still highly debated. Recent work has shown TRPC3 channels (Um et al., 2021) as well as an unidentified 1-(2,4-xylyl)guanidinium (XG)-sensitive current (Jehasse et al., 2021) also provide subthreshold depolarization that contributes to spontaneous firing in SNc DANs. NALCN, TRPC3, and the XG-sensitive current may work in concert to drive pacemaking in midbrain DANs. Future work will need to investigate the relative contributions of each of these conductances to pacemaking of DAN subpopulations.

Our results show that acute blockade of NALCN with Gd and L-703,606 in SNc neurons has little effect on pacemaking, which seems inconsistent with earlier results showing that genetic deletion of NALCN (NALCN cKO) abolishes pacemaking (Philippart and Khaliq, 2018). The reason for this discrepancy is unclear; however, it is possible that loss of NALCN from birth results in downregulation of other inward currents that drive depolarization and contribute to spontaneous firing, upregulation of potassium leak currents, and/or maladaptive changes throughout the development of the animal (Chan et al., 2007; Sinke et al., 2011; Lutas et al., 2016). Future experiments will need to examine changes to conductances that occur in NALCN cKO mice, including the effect of genetic deletion of NALCN in adult animals.

Last, previous studies have shown that reliance on HCN and L-type calcium channels for pacemaking varies across development (Chan et al., 2007; Branch et al., 2014). The experiments in this study were conducted in adult animals; however, it is possible that reliance on NALCN for pacemaking also changes across development. Future experiments should investigate how contribution of NALCN to pacemaking is potentially altered across development.

### Lowering extracellular calcium concentrations enhances firing of SNc dopaminergic neurons through potentiation of NALCN

The concentration of extracellular calcium can play a significant role in cellular excitability. Under physiological conditions, extracellular calcium concentrations range between 1.2 and 2 mM in the cerebrospinal fluid (Pye and Aber, 1982; Forsberg et al., 2019) and drop during normal synaptic transmission (King et al., 2001; Rusakov and Fine, 2003; Häusser et al., 2004). Such fluctuations in extracellular calcium concentrations have been shown to increase neuronal excitability, neurotransmitter release, synaptic transmission, and plasticity (Frankenhaeuser and Hodgkin, 1957; Lu et al., 2010; Jones and Smith, 2016; Philippart and Khaliq, 2018). Low extracellular calcium concentrations are associated with a variety of clinical disorders (Silver and Erecińska, 1990; Heinemann et al., 1992; Morris and Trippenbach, 1993; Nilsson et al., 1996).

Here, we showed that potentiation of NALCN with low (0.1 mM) extracellular calcium concentration speeds the rate of spontaneous firing of DANs. These data are consistent with previous work demonstrating that low extracellular calcium potentiates a nonselective cation current through NALCN (Lu et al., 2010; Philippart and Khaliq, 2018; Chua et al., 2020) and suggests that NALCN regulates firing of neurons when extracellular calcium fluctuates. By contrast, a recent study has suggested that NALCN is not directly involved in enhanced excitability with low calcium, instead suggesting that low calcium shifts the gating properties of voltage-gated sodium channels with minor contributions from NALCN (Martiszus et al., 2021). If the latter were the case, lowering extracellular calcium concentrations would have changed the firing rate of DANs independent of the presence of NALCN through charge screening. However, we found that lowering calcium had no effect on firing in both spontaneously active and silent cells in NALCN cKO mice. Therefore, although changes in charge screening with low extracellular calcium can increase the amplitude of subthreshold voltage-gated sodium conductances by shifting its voltage dependence, our data suggest that the dominant effect of lowering calcium in the DANs is to speed firing by increasing current through NALCN channels. Therefore, our results add to a body of work suggesting that NALCN is a part of the mechanism through which calcium regulates neuronal excitability in midbrain DANs. However, it will be important for future studies to examine the specific mechanisms by which low calcium regulates NALCN and neuronal excitability.

### Pharmacological blockade of NALCN using Gd

Previous literature has shown calcium channel blockade following application of Gd (Bourne and Trifaró, 1982; Yang and Sachs, 1989; Lacampagne et al., 1994). Although it is possible that blockade of calcium channels and calcium-activated potassium channels affects the pacemaker rate of midbrain DANs, application of Gd had no effect on firing of spontaneously active medVTA DANs in NALCN cKO mice in our control experiments. These data provide robust evidence that the effects we see on pacemaker activity are primarily because of inhibition of NALCN. Additionally, we replicated our findings with Gd using L-703,606 in all DAN subpopulations. L-703,606 has been shown to be specific for NALCN at concentrations up to 10 μM in DANs (Hahn et al., 2020). We found no difference in the effects of Gd and L-703,606 in any DAN subpopulation we tested. Together, these data suggest the dominant effect of Gd application is inhibition of NALCN and likely not because of complex nonspecific effects of Gd.

### Functional significance

DAN subpopulations play distinct roles in different neuropsychiatric conditions and behaviors. SNc DANs are implicated in movement disorders, such as Parkinson’s disease (Gonzalez-Rodiguez et al., 2020), suggesting a critical role of regular, robust pacemaking in these cells, while VTA DANs are implicated in disorders, such as depression and addiction (Nestler and Carlezon, 2006; Bromberg-Martin et al., 2010). Here we show that the contribution of NALCN to pacemaking of DAN subpopulations differs along the medial-lateral axis. Interestingly, there is also a medial-lateral gradient in the signaling of value and salience by DANs, although it is worth noting that this gradient is not consistent across all behaviors (Schultz et al., 1997; Saddoris et al., 2015; Han et al., 2017; de Jong et al., 2019; Li et al., 2019; Yuan et al., 2019; Cai et al., 2020; Kutlu et al., 2021). Knowledge of differences in the mechanism of pacemaking paves the way for the development of new pharmacology that selectively targets these dopaminergic subpopulations, which play distinct roles in behavior and neuropsychiatric disorders.

## References

[B1] Anderegg A, Poulin JF, Awatramani R (2015) Molecular heterogeneity of midbrain dopaminergic neurons: moving toward single cell resolution. FEBS Lett 589:3714–3726. 10.1016/j.febslet.2015.10.022 26505674 PMC4679573

[B2] Aransay A, Rodríguez-López C, García-Amado M, Clascá F, Prensa L (2015) Long-range projection neurons of the mouse ventral tegmental area: a single-cell axon tracing analysis. Front Neuroanat 9:59. 10.3389/fnana.2015.00059 26042000 PMC4436899

[B3] Baik JH (2020) Stress and the dopaminergic reward system. Exp Mol Med 52:1879–1890. 10.1038/s12276-020-00532-4 33257725 PMC8080624

[B4] Beier KT, Steinberg EE, DeLoach KE, Xie S, Miyamichi K, Schwarz L, Gao XJ, Kremer EJ, Malenka RC, Luo L (2015) Circuit architecture of VTA dopamine neurons revealed by systematic input–output mapping. Cell 162:622–634. 10.1016/j.cell.2015.07.015 26232228 PMC4522312

[B5] Bourne GW, Trifaró JM (1982) The gadolinium ion: a potent blocker of calcium channels and catecholamine release from cultured chromaffin cells. Neuroscience 7:1615–1622. 10.1016/0306-4522(82)90019-7 6289176

[B6] Branch SY, Sharma R, Beckstead MJ (2014) Aging decreases L-type calcium channel currents and pacemaker firing fidelity in substantia nigra dopamine neurons. J Neurosci 34:9310–9318. 10.1523/JNEUROSCI.4228-13.2014 25009264 PMC4087208

[B7] Bromberg-Martin ES, Matsumoto M, Hikosaka O (2010) Dopamine in motivational control: rewarding, aversive, and alerting. Neuron 68:815–834. 10.1016/j.neuron.2010.11.022 21144997 PMC3032992

[B8] Cai LX, Pizano K, Gundersen GW, Hayes CL, Fleming WT, Holt S, Cox JM, Witten IB (2020) Distinct signals in medial and lateral VTA dopamine neurons modulate fear extinction at different times. Elife 9:e54936. 10.7554/Elife.54936 32519951 PMC7363446

[B9] Chan CS, Guzman JN, Ilijic E, Mercer JN, Rick C, Tkatch T, Meredith GE, Surmeier DJ (2007) ‘Rejuvenation’ protects neurons in mouse models of Parkinson’s disease. Nature 447:1081–1086. 10.1038/nature05865 17558391

[B10] Chua HC, Wulf M, Weidling C, Rasmussen LP, Pless SA (2020) The NALCN channel complex is voltage sensitive and directly modulated by extracellular calcium. Sci Adv 6:eaaz3154. 10.1126/sciadv.aaz3154 32494638 PMC7182417

[B11] Costa KM, Schenkel D, Roeper J (2021) Sex-dependent alterations in behavior, drug responses and dopamine transporter expression in heterozygous DAT-Cre mice. Sci Rep 11:3334. 10.1038/s41598-021-82600-x 33558587 PMC7870653

[B12] Crill WE (1996) Persistent sodium current in mammalian central neurons. Annu Rev Physiol 58:349–362. 10.1146/annurev.ph.58.030196.002025 8815799

[B13] de Jong JW, Afjei SA, Dorocic IP, Peck JR, Liu C, Kim CK, Tian L, Deisseroth K, Lammel S (2019) A neural circuit mechanism for encoding aversive stimuli in the mesolimbic dopamine system. Neuron 101:133–151.e7. 10.1016/j.neuron.2018.11.005 30503173 PMC6317997

[B14] Flourakis M, Kula-Eversole E, Hutchison AL, Han TH, Aranda K, Moose DL, White KP, Dinner AR, Lear BC, Ren D, Diekman CO, Raman IM, Allada R (2015) A conserved bicycle model for circadian clock control of membrane excitability. Cell 162:836–848. 10.1016/j.cell.2015.07.036 26276633 PMC4537776

[B15] Ford NC, Ren D, Baccei ML (2018) NALCN channels enhance the intrinsic excitability of spinal projection neurons. Pain 159:1719–1730. 10.1097/j.pain.0000000000001258 29746349 PMC6095712

[B16] Forsberg M, Seth H, Björefeldt A, Lyckenvik T, Andersson M, Wasling P, Zetterberg H, Hanse E (2019) Ionized calcium in human cerebrospinal fluid and its influence on intrinsic and synaptic excitability of hippocampal pyramidal neurons in the rat. J Neurochem 149:452–470. 10.1111/jnc.14693 30851210

[B17] Frankenhaeuser B, Hodgkin AL (1957) The action of calcium on the electrical properties of squid axons. J Physiol 137:218–244. 10.1113/jphysiol.1957.sp005808 13449874 PMC1362975

[B18] Gantz SC, Ford CP, Morikawa H, Williams JT (2018) The evolving understanding of dopamine neurons in the substantia nigra and ventral tegmental area. Annu Rev Physiol 80:219–241. 10.1146/annurev-physiol-021317-121615 28938084

[B19] Gonzalez-Rodiguez P, Zampese E, Surmeier DJ (2020) Selective neuronal vulnerability in Parkinson’s disease. Prog Brain Res 252:61–89. 32247375 10.1016/bs.pbr.2020.02.005

[B20] Grace AA, Bunney BS (1984) The control of firing pattern in nigral dopamine neurons: single spike firing. J Neurosci 4:2866–2876. 10.1523/JNEUROSCI.04-11-02866.1984 6150070 PMC6564731

[B21] Guzman JN, Sánchez-Padilla J, Chan CS, Surmeier DJ (2009) Robust pacemaking in substantia nigra dopaminergic neurons. J Neurosci 29:11011–11019. 10.1523/JNEUROSCI.2519-09.2009 19726659 PMC2784968

[B22] Hage TA, Sun Y, Khaliq ZM (2016) Electrical and Ca^2+^ signaling in dendritic spines of substantia nigra dopaminergic neurons. Elife 5:e13905. 10.7554/Elife.13905 27163179 PMC4900803

[B23] Hahn S, Kim SW, Um KB, Kim HJ, Park MK, (2020) N-benzhydryl quinuclidine compounds are a potent and Src kinase-independent inhibitor of NALCN channels. Br J Pharmacol 177:3795–3810. 10.1111/bph.15104 32436268 PMC7402281

[B24] Hahn S, Um KB, Kim SW, Kim HJ, Park MK (2023) Proximal dendritic localization of NALCN channels underlies tonic and burst firing in nigral dopaminergic neurons. J Physiol 601:171–193. 10.1113/JP283716 36398712

[B25] Han X, Jing M, Zhao T, Wu N, Song R, Li J (2017) Role of dopamine projections from ventral tegmental area to nucleus accumbens and medial prefrontal cortex in reinforcement behaviors assessed using optogenetic manipulation. Metab Brain Dis 32:1491–1502. 10.1007/s11011-017-0023-3 28523568

[B26] Häusser M, Raman IM, Otis T, Smith SL, Nelson A, du Lac S, Loewenstein Y, Mahon S, Pennartz C, Cohen I, Yarom Y (2004) The beat goes on: spontaneous firing in mammalian neuronal microcircuits. J Neurosci 24:9215–9219. 10.1523/JNEUROSCI.3375-04.2004 15496653 PMC6730100

[B27] Heinemann SH, Terlau H, Stühmer W, Imoto K, Numa S (1992) Calcium channel characteristics conferred on the sodium channel by single mutations. Nature 356:441–443. 10.1038/356441a0 1313551

[B28] Ikemoto S (2007) Dopamine reward circuitry: two projection systems from the ventral midbrain to the nucleus accumbens-olfactory tubercle complex. Brain Res Rev 56:27–78. 10.1016/j.brainresrev.2007.05.004 17574681 PMC2134972

[B29] Jehasse K, Massotte L, Hartmann S, Vitello R, Ringlet S, Vitello M, Chua HC, Pless SA, Engel D, Liégeois JF, Lakaye B, Roeper J, Seutin V (2021) The gating pore blocker 1-(2,4-xylyl)guanidinium selectively inhibits pacemaking of midbrain dopaminergic neurons. Neuropharmacology 197:108722. 10.1016/j.neuropharm.2021.108722 34273387

[B30] Johnson SW, North RA (1992) Two types of neurone in the rat ventral tegmental area and their synaptic inputs. J Physiol 450:455–468. 10.1113/jphysiol.1992.sp019136 1331427 PMC1176131

[B31] Jones BL, Smith SM (2016) Calcium-sensing receptor: a key target for extracellular calcium signaling in neurons. Front Physiol 7:116. 10.3389/fphys.2016.00116 27065884 PMC4811949

[B32] Kang Y, Chen L (2022) Structure and mechanism of NALCN-FAM155A-UNC79-UNC80 channel complex. Nat Commun 13:2639. 10.1038/s41467-022-30403-7 35550517 PMC9098444

[B33] Kang Y, Kitai ST (1993) Calcium spike underlying rhythmic firing in dopaminergic neurons of the rat substantia nigra. Neurosci Res 18:195–207. 10.1016/0168-0102(93)90055-u 7907413

[B34] Kang Y, Wu JX, Chen L (2020) Structure of voltage-modulated sodium-selective NALCN-FAM155A channel complex. Nat Commun 11:6199. 10.1038/s41467-020-20002-9 33273469 PMC7712781

[B35] Khaliq ZM, Bean BP (2010) Pacemaking in dopaminergic ventral tegmental area neurons: depolarizing drive from background and voltage-dependent sodium conductances. J Neurosci 30:7401–7413. 10.1523/JNEUROSCI.0143-10.2010 20505107 PMC2892804

[B36] King RD, Wiest MC, Montague PR (2001) Extracellular calcium depletion as a mechanism of short-term synaptic depression. J Neurophysiol 85:1952–1959. 10.1152/jn.2001.85.5.1952 11353012

[B38] Koyama S, Kanemitsu Y, Weight F (2005) Spontaneous activity and properties of two types of principal neurons from the ventral tegmental area of rat. J Neurophysiol 93:3282–3293. 10.1152/jn.00776.2004 15659533

[B39] Kramer DJ, Risso D, Kosillo P, Ngai J, Bateup HS (2018) Combinatorial expression of Grp and Neurod6 defines dopamine neuron populations with distinct projection patterns and disease vulnerability. eNeuro 5:ENEURO.0152-18.2018. 10.1523/ENEURO.0152-18.2018 PMC6104179 30135866

[B40] Kutlu MG, Zachry JE, Melugin PR, Cajigas SA, Chevee MF, Kelley SJ, Kutlu B, Tian L, Siciliano CA, Calipari ES (2021) Dopamine release in the nucleus accumbens core signals perceived saliency. Curr Biol 31:4748–4761.e8. 10.1016/j.cub.2021.08.052 34529938 PMC9084920

[B41] Lacampagne A, Gannier F, Argibay J, Garnier D, Le Guennec JY (1994) The stretch-activated ion channel blocker gadolinium also blocks L-type calcium channels in isolated ventricular myocytes of the guinea-pig. Biochim Biophys Acta 1191:205–208. 10.1016/0005-2736(94)90250-x 8155676

[B42] Lammel S, Hetzel A, Häckel O, Jones I, Liss B, Roeper J (2008) Unique properties of mesoprefrontal neurons within a dual mesocorticolimbic dopamine system. Neuron 57:760–773. 10.1016/j.neuron.2008.01.022 18341995

[B43] Lammel S, Lim BK, Malenka RC (2014) Reward and aversion in a heterogeneous midbrain dopamine system. Neuropharmacology 76:351–359. 10.1016/j.neuropharm.2013.03.019 23578393 PMC3778102

[B44] Lerner TN, Shilyansky C, Davidson TJ, Evans KE, Beier KT, Zalocusky KA, Crow AK, Malenka RC, Luo L, Tomer R, Deisseroth K (2015) Intact-brain analyses reveal distinct information carried by SNc dopamine subcircuits. Cell 162:635–647. 10.1016/j.cell.2015.07.014 26232229 PMC4790813

[B45] Li H, Illenberger JM, Cranston MN, Mactutus CF, McLaurin KA, Harrod SB, Booze RM (2019) Posterior ventral tegmental area-nucleus accumbens shell circuitry modulates response to novelty. PLoS One 14:e0213088. 10.1371/journal.pone.0213088 30835756 PMC6400398

[B46] Lu B, Su Y, Das S, Liu J, Xia J, Ren D (2007) The neuronal channel NALCN contributes resting sodium permeability and is required for normal respiratory rhythm. Cell 129:371–383. 10.1016/j.cell.2007.02.041 17448995

[B47] Lu B, Su Y, Das S, Wang H, Wang Y, Liu J, Ren D (2009) Peptide neurotransmitters activate a cation channel complex of NALCN and UNC-80. Nature 457:741–744. 10.1038/nature07579 19092807 PMC2810458

[B48] Lu B, Zhang Q, Wang H, Wang Y, Nakayama M, Ren D (2010) Extracellular calcium controls background current and neuronal excitability via an UNC79-UNC80-NALCN cation channel complex. Neuron 68:488–499. 10.1016/j.neuron.2010.09.014 21040849 PMC2987630

[B49] Lutas A, Lahmann C, Soumillon M, Yellen G (2016) The leak channel NALCN controls tonic firing and glycolytic sensitivity of substantia nigra pars reticulata neurons. Elife 5:e15271. 10.7554/Elife.15271 27177420 PMC4902561

[B50] Margolis EB, Lock H, Hjelmstad GO, Fields HL (2006) The ventral tegmental area revisited: is there an electrophysiological marker for dopaminergic neurons? J Physiol 577:907–924. 10.1113/jphysiol.2006.117069 16959856 PMC1890372

[B51] Martiszus BJ, Tsintsadze T, Chang W, Smith SM (2021) Enhanced excitability of cortical neurons in low-divalent solutions is primarily mediated by altered voltage-dependence of voltage-gated sodium channels. Elife 10:e67914. 10.7554/Elife.67914 33973519 PMC8163501

[B52] Mercuri NB, Bonci A, Calabresi P, Stefani A, Bernardi G (1995) Properties of the hyperpolarization-activated cation current Ih in rat midbrain dopaminergic neurons. Eur J Neurosci 7:462–469. 10.1111/j.1460-9568.1995.tb00342.x 7773443

[B53] Morris ME, Trippenbach T (1993) Changes in extracellular [K^+^] and [Ca^2+^] induced by anoxia in neonatal rabbit medulla. Am J Physiol 264:R761–R769. 10.1152/ajpregu.1993.264.4.R761 8476118

[B54] Nedergaard S, Greenfield SA (1992) Sub-populations of pars compacta neurons in the substantia nigra: the significance of qualitatively and quantitatively distinct conductances. Neuroscience 48:423–437. 10.1016/0306-4522(92)90502-s 1603327

[B55] Nestler EJ, Carlezon WA (2006) The mesolimbic dopamine reward circuit in depression. Biol Psychiatry 59:1151–1159. 10.1016/j.biopsych.2005.09.018 16566899

[B56] Neuhoff H, Neu A, Liss B, Roeper J (2002) I(h) channels contribute to the different functional properties of identified dopaminergic subpopulations in the midbrain. J Neurosci 22:1290–1302. 10.1523/JNEUROSCI.22-04-01290.2002 11850457 PMC6757558

[B57] Nilsson P, Laursen H, Hillered L, Hansen AJ (1996) Calcium movements in traumatic brain injury: the role of glutamate receptor-operated ion channels. J Cereb Blood Flow Metab 16:262–270. 10.1097/00004647-199603000-00011 8594058

[B58] Philippart F, Destreel G, Merino-Sepúlveda P, Henny P, Engel D, Seutin V (2016) Differential somatic Ca^2+^ channel profile in midbrain dopaminergic neurons. J Neurosci 36:7234–7245. 10.1523/JNEUROSCI.0459-16.2016 27383597 PMC6705535

[B59] Philippart F, Khaliq ZM (2018) Gi/o protein-coupled receptors in dopamine neurons inhibit the sodium leak channel NALCN. Elife 7:e40984. 10.7554/Elife.40984 30556810 PMC6305199

[B60] Poulin JF, Caronia G, Hofer C, Cui Q, Helm B, Ramakrishnan C, Chan CS, Dombeck D, Deisseroth K, Awatramani R (2018) Mapping projections of molecularly defined dopamine neuron subtypes using intersectional genetic approaches. Nat Neurosci 21:1260–1271. 10.1038/s41593-018-0203-4 30104732 PMC6342021

[B61] Puopolo M, Raviola E, Bean BP (2007) Roles of subthreshold calcium current and sodium current in spontaneous firing of mouse midbrain dopamine neurons. J Neurosci 27:645–656. 10.1523/JNEUROSCI.4341-06.2007 17234596 PMC6672803

[B62] Pye IF, Aber GM (1982) Interrelations between cerebrospinal fluid and plasma inorganic ions and glucose in patients with chronic renal failure. J Clin Pathol 35:631–637. 10.1136/jcp.35.6.631 7085915 PMC497739

[B63] Rusakov DA, Fine A (2003) Extracellular Ca^2+^ depletion contributes to fast activity-dependent modulation of synaptic transmission in the brain. Neuron 37:287–297. 10.1016/s0896-6273(03)00025-4 12546823 PMC3375894

[B64] Saddoris MP, Cacciapaglia F, Wightman RM, Carelli RM (2015) Differential dopamine release dynamics in the nucleus accumbens core and shell reveal complementary signals for error prediction and incentive motivation. J Neurosci 35:11572–11582. 10.1523/JNEUROSCI.2344-15.2015 26290234 PMC4540796

[B65] Schultz W, Dayan P, Montague PR (1997) A neural substrate of prediction and reward. Science 275:1593–1599. 10.1126/science.275.5306.1593 9054347

[B66] Seutin V, Massotte L, Renette MF, Dresse A (2001) Evidence for a modulatory role of Ih on the firing of a subgroup of midbrain dopamine neurons. Neuroreport 12:255–258. 10.1097/00001756-200102120-00015 11209930

[B67] Shi Y, Abe C, Holloway BB, Shu S, Kumar NN, Weaver JL, Sen J, Perez-Reyes E, Stornetta RL, Guyenet PG, Bayliss DA (2016) Nalcn is a ‘leak’ sodium channel that regulates excitability of brainstem chemosensory neurons and breathing. J Neurosci 36:8174–8187. 10.1523/JNEUROSCI.1096-16.2016 27488637 PMC4971364

[B68] Silver IA, Erecińska M (1990) Intracellular and extracellular changes of [Ca^2+^] in hypoxia and ischemia in rat brain in vivo. J Gen Physiol 95:837–866. 10.1085/jgp.95.5.837 2163431 PMC2216343

[B69] Sinke AP, Caputo C, Tsaih SW, Yuan R, Ren D, Deen PM, Korstanje R (2011) Genetic analysis of mouse strains with variable serum sodium concentrations identifies the Nalcn sodium channel as a novel player in osmoregulation. Physiol Genomics 43:265–270. 10.1152/physiolgenomics.00188.2010 21177381 PMC3068516

[B70] Um KB, Hahn S, Kim SW, Lee YJ, Birnbaumer L, Kim HJ, Park MK (2021) TRPC3 and NALCN channels drive pacemaking in substantia nigra dopaminergic neurons. Elife 10:e70920. 10.7554/Elife.70920 34409942 PMC8456572

[B71] Yang XC, Sachs F (1989) Block of stretch-activated ion channels in *Xenopus* oocytes by gadolinium and calcium ions. Science 243:1068–1071. 10.1126/science.2466333 2466333

[B72] Yeh SY, Huang WH, Wang W, Ward CS, Chao ES, Wu Z, Tang B, Tang J, Sun JJ, Esther van der Heijden M, Gray PA, Xue M, Ray RS, Ren D, Zoghbi HY (2017) Respiratory network stability and modulatory response to substance P require Nalcn. Neuron 94:294–303.e4. 10.1016/j.neuron.2017.03.024 28392070 PMC5702257

[B73] Yuan L, Dou YN, Sun YG (2019) Topography of reward and aversion encoding in the mesolimbic dopaminergic system. J Neurosci 39:6472–6481. 10.1523/JNEUROSCI.0271-19.2019 31217328 PMC6697397

